# NOX-NOS crosstalk in the liver-brain axis: Novel insights for redox regulation and neurodegenerative diseases

**DOI:** 10.1016/j.redox.2025.103807

**Published:** 2025-08-06

**Authors:** Sang-Seop Lee, Yung-Choon Yoo

**Affiliations:** Department of Microbiology, College of Medicine, Konyang University, Daejeon, 32992, South Korea

**Keywords:** Liver-brain axis, NADPH oxidase, Nitric oxide synthase, NOX-NOS crosstalk, Neurodegenerative diseases, Oxidation-reduction reaction

## Abstract

The liver-brain axis is an emerging concept linking liver dysfunction and brain disease. Hepatic metabolic abnormalities induce systemic oxidative stress and endothelial dysfunction, which contribute to central nervous system (CNS) inflammation and neurodegeneration. Redox regulation plays a key role in the liver-brain axis, with NADPH oxidase (NOX) and nitric oxide synthase (NOS) being involved in the generation of various reactive oxygen species (ROS) and reactive nitrogen species (RNS), respectively, thereby inducing oxidative stress and disrupting the NADPH/NADP balance. Dysregulation of NOX-NOS cross-signaling not only amplifies oxidative stress, but also disrupts endothelial homeostasis and exacerbates neuroinflammation, leading to progressive neurodegeneration. For instance, reactive carbonyl species such as methylglyoxal (MGO) and acrolein can upregulate NOX isoforms and stimulate NLRP (NOD like receptor protein) inflammasomes activation, illustrating disease-relevant links between hepatic redox imbalance and CNS pathology. Mechanistically, superoxide (O_2_•^-^) generated by NOX readily reacts with nitric oxide (•NO) derived from NOS to form peroxynitrite (ONOO^−^), a highly reactive oxidant that exacerbates vascular and neuronal injury. Despite extensive research on NOX and NOS, their interactive contributions to redox imbalance and the progression of neurodegenerative diseases remain poorly understood. In this review, we introduce the NOX-NOS axis as a key regulator of the liver-brain axis, and highlight the roles of NOX and NOS in linking hepatic metabolic dysfunction to central nervous system pathology through intermediary metabolites in the exacerbation of neuroinflammation and oxidative stress. We also explore therapeutic strategies targeting NOX-NOS interactions, including selective NOX inhibitors, NOS modulators, and redox homeostasis regulators, providing new insights into redox regulation and the management of metabolic neurodegenerative diseases.

## Graphical abstract

**Figure:**
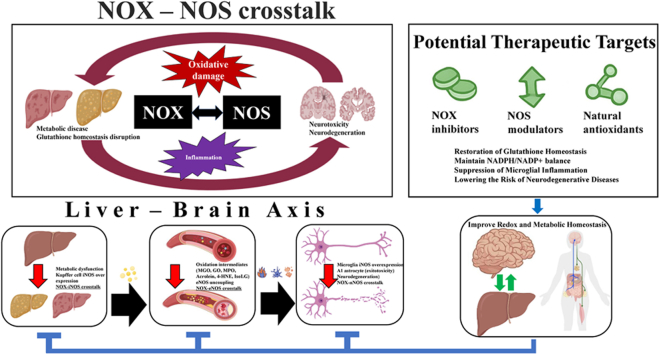

## 1 Introduction

Oxidative stress and inflammation are deeply interconnected processes that play pivotal roles in the pathogenesis of neuro-degenerative diseases, including Alzheimer's disease (AD), Parkinson's disease (PD), and amyotrophic lateral sclerosis (ALS) [[1], [2], [3]]. The brain, due to its high metabolic activity and limited antioxidant defenses, is particularly vulnerable to oxidative damage [4]. This damage contributes to neuropathological hallmarks such as amyloid plaques and neurofibrillary tangles in AD, as well as excitotoxicity and metabolic dysfunction across a wide spectrum of neurodegenerative conditions [5,6]. Furthermore, interactions between microglia and astrocytes, mediated by extracellular vesicles (EVs), have been implicated in driving neuroinflammation and neuronal dysfunction [7]. Although oxidative stress and inflammation are known to be established drivers of neurodegenerative diseases, recent findings suggest that systemic factors, such as liver function, may serve as key modulators of these processes [8,9].

As the central organ of redox homeostasis, the liver plays a critical role in maintaining antioxidant defenses through glutathione (GSH)-mediated mechanisms and regulatory pathways such as nuclear factor erythroid 2-related factor 2 (NRF2)/heme oxygenase 1 (HO1) and NAD(P)H quinone dehydrogenase 1 (NQO1) [[10], [11], [12], [13]]. Liver dysfunction disrupts endothelial homeostasis by causing metabolic system collapse through exacerbation of systemic oxidative stress. Consequently, this leads to CNS dysfunction and ultimately contributes to the development of neurodegenerative diseases. This series of interactions aligns well with the concept of the liver-brain axis [14].

A multidisciplinary perspective on the organ-liver axis, including the liver-brain axis, and the interactions between these organs has attracted attention as a new approach to explore the etiology of various diseases and develop treatments. [15,16]. These concepts should be approached through a systematic methodology based on traditional methods, but it is only recently that this has begun to be fully applied to life sciences. In particular, the liver-brain axis has received renewed attention in recent years in the fields of neurological and metabolic diseases and is emerging as a cutting-edge field of inter-organ axis research [17]. From this perspective of the liver-brain axis, it is certain that metabolic and redox regulation serve as important mechanisms linking systemic oxidative stress and neurodegeneration [18].

**Fig. 1:**
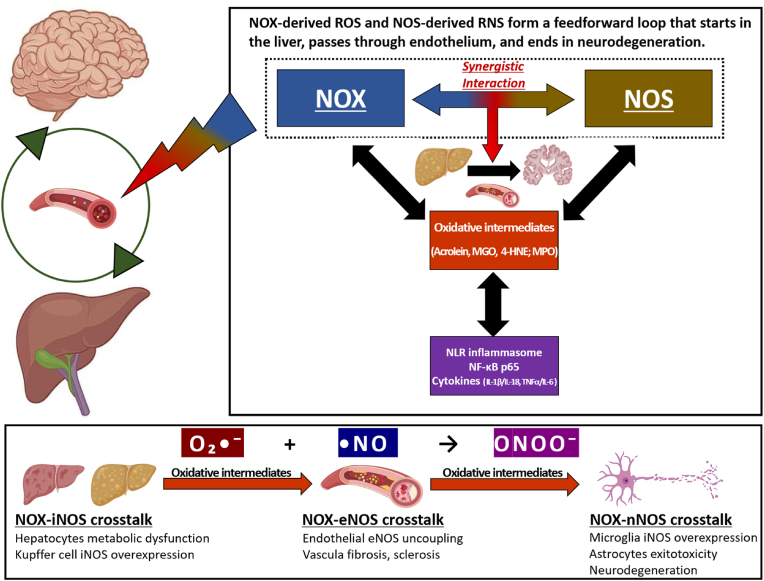
Synergistic interplay between NOX- and NOS-derived reactive species along the liver–brain axis.

NADPH oxidase (NOX) and nitric oxide synthase (NOS) are central producers of reactive oxygen species (ROS) and reactive nitrogen species (RNS), respectively, mediating a wide range of oxidative damage and inflammatory responses. However, while there are many existing reports that have investigated NOX and NOS individually, studies focusing on the interaction between them are relatively limited [19,20]. Similarly, although there are scientific papers investigating the crosstalk between ROS and RNS [21], there are •NO reports yet describing the broader metabolic-oxidative-inflammatory context focusing on the intimate interplay between NOX and NOS. Against this background, this review describes that NOX and NOS are key regulators of the liver-brain axis (Fig. 1) and regulate pathways that can cause CNS dysfunction by disrupting redox regulation and metabolic homeostasis. Moreover, it highlights the synergistic role of the NOX-NOS axis in the initiation and exacerbation of neuroinflammation and oxidative stress. Finally, we explore the therapeutic potential of targeting NOX and NOS to mitigate oxidative damage and inflammation, thereby providing new insights into the management of neurodegenerative diseases.

This figure illustrates a feedforward loop in which NOX-derived ROS and NOS-derived RNS cooperatively propagate redox and inflammatory stress from the liver, through the endothelium, to the brain. The mechanisms illustrated in this figure are described in detail in Sections 2, 3.3 of the main text.

NOX isoforms and NOS isoforms generate O_2_•^-^ and •NO, which combine to form peroxynitrite (ONOO^−^), a central effector of NOX–NOS crosstalk. Under NOX-induced oxidative stress, NOS activity becomes dysregulated, often leading to excessive •NO production and enhanced ONOO^−^ formation.

Reactive oxidative intermediates, including methylglyoxal (MGO), 4-hydroxynonenal (4-HNE), acrolein, and myeloperoxidase (MPO), mediate signal amplification across tissues and contribute to endothelial dysfunction, inflammasome activation, and neurodegeneration (Section 2.2.2). These intermediates link hepatic redox imbalance to activation of the NLRP inflammasomes and NF-κB p65, leading to the release of key proinflammatory cytokines (IL-1β, IL-18, TNF-α, and IL-6).

## 2 NOX and NOS: structural and functional overview

### 2.1 NOX and NOS isoforms and mechanisms

#### 2.1.1 NOX isoforms and mechanisms

The NOX family comprises seven isoforms NOX1 to NOX5, DUOX1, and DUOX2 that catalyze the production of ROS using NADPH as an electron donor (Table 1). Oxidative damage caused by O_2_•^-^/H_2_O_2_ is primarily regulated by NOX, which acts as an upstream modulator [22].

**Table 1:** Summary of biological functions across NOX isoforms.

Feature	NOX1	NOX2	NOX3	NOX4	NOX5	DUOX1	DUOX2
**Expression Location**	**Plasma membrane, endosomes**	**Plasma membrane, phagosomes**	**Plasma membrane, inner ear**	**Mitochondria, endoplasmic reticulum**	**Plasma membrane.** **Rodents lack NOX5, limiting preclinical data**	**Plasma membrane, thyroid tissue**	**Plasma membrane, respiratory epithelium**
**Major Distribution**	**Colon, vascular smooth muscle**	**Neutrophils, macrophages, microglia**	**Inner ear (cochlea)**	**Kidney, heart, vascular smooth muscle**	**Testis, vascular smooth muscle, endothelial cells**	**Thyroid, airway epithelium**	**Salivary glands, airway epithelium**
**Binding Units**	**p22phox, NOXO1, NOXA1, Rac1**	**p22phox, p47** **phox, p67phox, p40phox, Rac1**	**p22phox, p47phox, NOXO1, Rac1**	**p22phox, Poldip2**	**EF-hand Ca^2+^-binding**	**DUOXA1, EF-hand Ca^2+^-binding**	**DUOXA2, EF-hand Ca^2+^-binding**
**Action in Liver-Brain Axis**	**Kupffer cells, astrocytes; promotes inflammation and oxidative stress**	**Impairment of Kupffer cell function, exacerbates neuroinflammation**	**Limited evidence in liver-brain axis-related diseases**	**Impairs astrocyte and hepatocyte metabolism, contributing to neurodegeneration**	**Linked to endothelial dysfunction and vascular oxidative stress**	**Minimal evidence in liver-brain axis disorders**	
**Related Pathways in Liver-Brain Axis**	**Rac1-NOX1 superoxide primes Kupffer→microglial cytokine axis**	**Hepatic NOX2–ROS may trigger brain NLRP3 activation via systemic circulation.**	**Limited but potential involvement of NOX3 in liver–brain axis–associated redox signaling.**	**NOX4-derived H_2_O_2_ from mitochondria/ER drives hepatocyte and astrocyte metabolic stress**	**Ca^2+^-triggered endothelial ROS disrupts BBB integrity**	**DUOX-derived H_2_O_2_ may act as a secondary amplifier of redox stress in the liver–brain axis, particularly under chronic inflammatory stimuli.**	
**Refs**	[19,20,23,[24], [25], [26],[27], [28], [29], [30]]	[19,20,[24], [25], [26],[27], [28], [29],^31^,^32^]	[19,20,[24], [25], [26],[27], [28], [29],^33^]	[19,20,23,[24], [25], [26],^34^,[27], [28], [29],^35^,^36^]	[19,20,[24], [25], [26], [37],[27], [28], [29],^38^]	[19,20,[24], [25], [26],[27], [28], [29],^39^,^40^]	[19,20,[24], [25], [26],[27], [28], [29],^39^,^40^]

Metabolic disorders such as obesity, diabetes, and fatty liver establish a systemic milieu that is particularly prone to redox imbalance, thereby amplifying the detrimental effects of ROS. While all NOX isoforms may contribute to oxidative dysregulation under such conditions, NOX2 and NOX4 are especially implicated in liver–brain axis dysfunction. NOX2, enriched in Kupffer cells and microglia, facilitates inflammatory propagation through systemic ROS signaling, whereas NOX4-derived H_2_O_2_ disrupts mitochondrial and ER function in hepatocytes and astrocytes, promoting neurodegeneration [[41], [42], [43], [44], [45]]. Although DUOX1/2 can directly produce H_2_O_2_ via their peroxidase-like domains, their contribution to NOX–NOS crosstalk appears minimal due to limited superoxide generation and restricted expression in liver–brain axis–relevant tissues. These isoform-specific actions not only intensify oxidative stress but also compromise redox-sensitive signaling pathways and endothelial integrity, underscoring the central role of NOX enzymes in metabolic and neuroinflammatory disease progression.

This table summarizes the key features of NOX isoforms including subcellular localization, tissue-specific expression, binding partners, and pathophysiological roles with a particular focus on their relevance to liver–brain axis disorders. Each row is organized to reflect a mechanistic sequence from cellular expression to disease outcomes, emphasizing how isoform-specific localization and assembly contribute to redox imbalance, neuroinflammation, and endothelial dysfunction.

Except for NOX4 and DUOX1/2, most NOX isoforms generate superoxide (O_2_•^-^), which is rapidly converted into membrane-permeable hydrogen peroxide (H_2_O_2_) by superoxide dismutase (SOD) [[23], [46], [47], [48], [49], [50]]. Excess accumulation of these ROS promotes oxidative injury via Fenton chemistry, lipid peroxidation, and protein oxidation, with NOX enzymes acting as key upstream regulators [[51], [52], [53], [54], [55], [56], [57]]. For instance, NOX2 expressed in microglial phagosomes and assembled via p47phox plays a pivotal role in propagating neuroinflammatory signaling.

Abbreviations: NOX, NADPH oxidase; DUOX, dual oxidase; ROS, reactive oxygen species; O_2_•^-^, superoxide anion; H_2_O_2_, hydrogen peroxide; SOD, superoxide dismutase; ER, endoplasmic reticulum; BBB, blood–brain barrier; EF-hand, calcium-binding domain; NLRP3, NOD like receptor protein 3; Rac1, Ras-related C3 botulinum toxin substrate 1; phox, phagocyte oxidase; NOXA1, NADPH oxidase activator 1; Poldip2, polymerase delta-interacting protein 2.

#### 2.1.2 NOS isoforms and mechanisms

Nitric oxide synthases (NOS) are central homeostatic enzymes that catalyze the oxygen-dependent conversion of l-arginine into nitric oxide (•NO), a multifunctional signaling molecule involved in vascular tone, insulin regulation, immune responses, gastrointestinal activity, and neurodevelopment [[58], [59], [60], [61], [62]]. NOS isoforms are categorized based on their predominant expression sites into neuronal NOS (nNOS or NOS–I), inducible NOS (iNOS or NOS-II), and endothelial NOS (eNOS or NOS-III) (Table 2).

**Table 2:** Summary of biological functions across NOS isoforms.

Feature	iNOS	nNOS	eNOS
**Expression Location**	**Cytoplasm**	**Neuronal cells, synaptic terminals**	**Endothelial cells, caveolae**
**Major Distribution**	**Macrophages, hepatocytes, astrocytes**	**Neurons, glial cells, smooth muscle**	**Vascular endothelial cells, lungs, heart**
**Binding Units**	**Calmodulin (CaM), tetrahydrobiopterin (BH4)**	**Calmodulin (CaM), tetrahydrobiopterin (BH4)**	**Calmodulin (CaM), tetrahydrobiopterin (BH4)**
**Action in Liver-Brain Axis**	**Kupffer cells, astrocytes; promotes inflammation and oxidative stress**	**Neurons, astrocytes; modulates neurotransmission and contributes to excitotoxicity**	**Endothelial cells; regulates vascular tone and protects against oxidative damage**
**Related Diseases in Liver-Brain Axis**	**Involved in neuroinflammation, excitotoxicity; contributes to liver fibrosis**	**Plays a role in neurodegenerative diseases via excitotoxicity**	**Dysfunction linked to endothelial damage, vascular oxidative stress**
**Refs**	[59,60,63]	[60,62,64]	[60,64,65]

From the perspective of the liver–brain axis, key inflammatory pathways such as TLR4–MyD88 signaling activate transcription factors including NF-κB, ATF4, and AP-1, leading to rapid iNOS induction and a surge in •NO/ONOO^−^ production [[63], [66], [67], [68]]. This cascade promotes the acquisition of a pro-inflammatory phenotype (commonly referred to as M1 polarization or, more recently, M1-like activation) in Kupffer cells and microglia, and facilitates NLRP3 inflammasome activation via nuclear translocation of acetylated NF-κB, ultimately promoting pyroptosis and neuroinflammation [69,70].

Oxidative stress driven by excessive iNOS activity or chronic NOX activation depletes intracellular BH_4_ and promotes the uncoupling of eNOS and nNOS, thereby creating a feedforward loop that amplifies ONOO^−^ production and exacerbates redox imbalance [71,72]. This feedforward mechanism highlights the core premise of NOX–NOS crosstalk in the liver–brain axis, wherein isoform-specific NOS dysregulation contributes to sustained oxidative damage, metabolic dysfunction, inflammation, and neurodegeneration [73,74].

This table summarizes the subcellular localization, tissue distribution, cofactor usage, and functional roles of the three major NOS isoforms (iNOS, nNOS, and eNOS). All isoforms depend on calmodulin (CaM) and tetrahydrobiopterin (BH_4_) for enzymatic activity, but differ in calcium sensitivity. eNOS and nNOS are activated by Ca^2+^ influx and are often referred to as constitutive NOS (cNOS) due to their basal expression and calcium-regulated activity under physiological conditions. In contrast, iNOS is highly inducible under inflammatory stimuli and contributes to oxidative stress and neuroinflammation via •NO and ONOO^−^ overproduction in Kupffer cells and astrocytes [[65], [75], [76], [77], [78]]. nNOS regulates neurotransmission but may exacerbate excitotoxicity in disease states, while eNOS protects endothelial function but becomes uncoupled under oxidative conditions. Together, isoform-specific dysregulation of NOS enzymes plays a central role in redox imbalance and inter-organ communication between the liver and brain.

Abbreviations: NOS, nitric oxide synthase; iNOS, inducible NOS; nNOS, neuronal NOS; eNOS, endothelial NOS; CaM, calmodulin; BH_4_, tetrahydrobiopterin; Kupffer cells, liver-resident macrophages; excitotoxicity, neuronal injury caused by excessive excitatory neurotransmitter signaling.

### 2.2 Reactive-species cascade in NOX and NOS

#### 2.2.1 Reactive intermediates that seed NOX/NOS crosstalk

ROS and RNS are representative reactive species responsible for oxidative damage; however, other species, such as reactive halogen species (RHS), reactive sulfur species (RSS), reactive carbonyl species (RCS), and reactive electrophilic species (RES), also play crucial roles in redox regulation [79,80].

RHS, mainly generated by myeloperoxidase (MPO), has functions similar to ROS and RNS, and participates in immune responses, inflammation, inflammasome activation, lipid peroxidation, and protein oxidation [[81], [82], [83], [84], [85], [86]]. It affects protein, lipid, and DNA signaling pathways, causing oxidative damage and leading to cell death.

RSS, a group of sulfur-based compounds that include hydrogen sulfide (H_2_S), persulfides, and polysulfides, is primarily synthesized from cysteine and is critical for maintaining redox homeostasis [[87], [88], [89], [90]]. RCS are generated both during lipid peroxidation—yielding aldehydes such as 4-hydroxy-2-nonenal (4-HNE) and malondialdehyde (MDA)—and during sugar oxidation, which produces dicarbonyls like methylglyoxal (MGO) and glyoxal (GO) [[34], [91], [92], [93], [94], [95], [96], [97], [98], [99], [100], [101], [102], [103], [104], [105], [106]]. Excessive RCS production contributes to the development of various diseases, including metabolic syndrome.

RES are highly reactive compounds that readily accept electrons, enabling them to rapidly interact with electron-rich regions of biomolecules, such as nucleophilic sites [[107], [108], [109], [110]]. Quinones are used as representative examples of RES, and other molecules including MDA, acrolein, and 4-HNE, are included in the scope of RCS while also being classified as RES. Highly reactive γ-keto-dialdehydes known as IsoLGs rapidly form Lys adducts that destabilize membrane and cytosolic proteins, potentiate NOX2-derived ROS, and diminish endothelial NO signalling, culminating in eNOS dysfunction [111,112].

Table 3 provides detailed examples of reactive intermediate metabolites, with a focus on those closely linked to NOX and NOS regulation. Classical molecules such as H_2_O_2_ and S-nitrosothiols are also included, as they directly modify NOX/NOS or their upstream regulators. These reactive intermediates serve as key molecular links that systemically amplify NOX–NOS crosstalk.

**Table 3:** Representative reactive intermediates that bridge NOX and NOS signalling.

Category/Source	Representative species	Formation pathway	Key mechanistic action on NOX/NOS axis	Disease/biomarker context	Ref
**ROS/Metal catalytic**	**H_2_O_2_**	O_2_•- dismutation (SOD); oxidase leakage	Diffuses across membranes; activates redox-kinases (such as Src) that prime NOX; up-regulates iNOS	Insulin resistance, endothelial dysfunction	[[50], [51], [52],[113], [114],^115^,^116^]
	**Fenton Fe^2+^/Fe^3+^**	H_2_O_2_ + Fe^2+^ → ·OH + Fe^3+^	·OH triggers lipid peroxidation → secondary RCS/RES; amplifies NOX	Ischaemia-reperfusion, ferroptosis	[53,57,117]
**RNS**	**S-nitrosothiols (such as GSNO, SSNO^−^)**	•NO + GSH/L-Cys (*trans*-nitrosylation)	S-nitrosylates eNOS (feedback inhibition) & p47 phox S-nitrosylation ⟶ ↓ROS; modulates PTP1B; GSNO: opposing effects in I/R (diabetic vs non-diabetic)	Vascular dysregulation, chronic inflammation	[21,118,119,120,[121], [122], [123], [124]]
	**Peroxynitrite (ONOO^−^)**	•NO (NOS) + O_2_•- (NOX)	Tyr-nitration; eNOS uncoupling; iNOS induction	Endothelial dysfunction, diabetic complications	[78,125,115]
**RHS — Peroxidase-derived halogen oxidants**	**MPO → HOCl**	H_2_O_2_ + Cl^−^ (MPO)	Oxidises Cys/Met; uncouples eNOS/nNOS; primes NOX2/4	MASLD, atherosclerosis	[126,127,117,128,129,130,131]
	**EPO → HOBr**	H_2_O_2_ + Br^−^ (EPO)	Halogenates lipids → IsoLG; activates NOX2	Asthma, eosinophilic GI disease	
	**LPO → HOBr**	H_2_O_2_ + Br^−^/I^−^ (LPO)	3-Br-Tyr formation; NOX2–p38 signalling	Airway infection, COPD	
**RES — Lipid-derived electrophiles**	**4-HNE**	ω-6 lipid peroxidation	NOX4→H_2_O_2_→↑OxStress; 4-HNE→AD/aging pathology	MASLD, AD	[83,34,132,[133], [134],^135^,[136], [137]]
	**4-ONE**	ω-6 lipid peroxidation	Michael adducts with Cys/His → inhibits NOS substrate access	Ischaemia, renal injury	[132,138]
	**Acrolein**	PUFA & threonine oxidation	Covalent adducts; up-regulates NOX2 + iNOS	Smoke exposure, stroke, neuroinflammation	[93,139,140,141,142]
	**Isolevuglandins (IsoLGs)**	ω-6 lipid peroxidation or COX-PGH_2_ rearrangement	Lys-εNH_2_ adducts that prime NOX2 activity; lower NO bioavailability → eNOS dysfunction	Hypertension, atherosclerosis, MASH, AD	[[111], [112], [143]]
**Oxidative lipid adducts**	**MDA-LDL**	RCS + LDL	CD36-NOX2 activation; eNOS uncoupling	Atherosclerosis, MetS	[54,144,138,145]
	**OxLDL**	ROS + LDL	LOX-1-NOX2 axis; iNOS surge	Atherosclerosis, MASLD	[144,146,145,147,136]
**RCS — Sugar-/glycolysis-derived dicarbonyls**	**MGO**	Triose-phosphate overflow	AGE/RAGE → NOX2/4; eNOS nitration	Diabetes, BBB leak	[[95], [96], [97], [98], [99],[108], [148],[149], [150]]
	**GO**	Glucose autoxidation	AGE/RAGE activation; NOX up-shift	MetS, retinopathy	[92,108]
	**3-DG**	Fructose degradation	AGE/RAGE →NOX1/4 ↑	Diabetic nephropathy	[92,151,152]
	**Glycolaldehyde**	Ascorbate/sugar oxidation	Protein cross-links; NOX2 activation	Cognitive decline, liver fibrosis	[153,154,155]

**Fig. 2:**
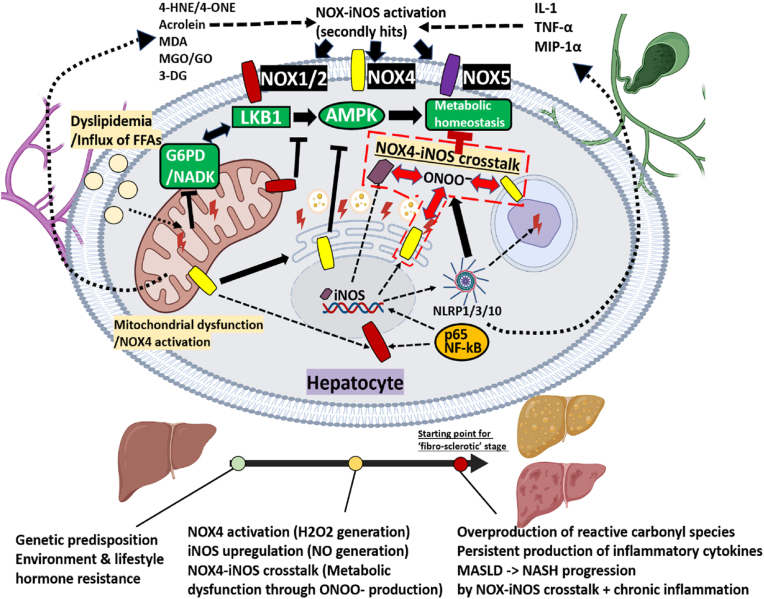
Metabolic “trigger” stage of the NOX-NOS crosstalk in liver–brain axis: NOX4-centred crosstalk initiates NOX–iNOS crosstalk and pro-inflammatory signalling. Dyslipidaemia and the influx of free fatty acids (FFAs) activate membrane NOX4 and suppress the LKB1 AMPK checkpoint, while reactive carbonyl precursors (4-HNE/4-ONE, acrolein, MDA, MGO/GO, 3-DG) provide a secondary hit that upregulates iNOS. NOX4-derived H_2_O_2_ and iNOS-derived •NO combine to form ONOO^−^, creating a NOX4-iNOS feed-forward loop (red dashed box) that depletes NADPH, oxidises BH_4_ and drives eNOS uncoupling. Mitochondrial dysfunction, ER stress, lysosomal permeabilisation and NLRP1/3/10 inflammasome activation amplify ROS/RNS release, while Kupffer cell cytokines (IL 1β, TNF α, MIP 1α) further stimulate NOX1/2/4/5 and iNOS. The cumulative effect creates a chronic metabolic inflammation milieu that drives the transition from MASLD to NASH (progress bar, bottom). The red arrow indicates the starting point of the fibrosclerotic stage shown in Fig. 3.

The table lists key small-molecule oxidants, electrophiles and dicarbonyls that (i) arise upstream of, or are amplified by, NADPH-oxidase (NOX) and nitric-oxide-synthase (NOS) activity and (ii) feed back to modulate either enzyme family. Species are grouped by chemical class: classical ROS, metal-catalysed ROS (Fenton chemistry), RNS, halogen oxidants (RHS), lipid-derived electrophiles (RES), oxidative lipid adducts, and sugar-derived dicarbonyl RCS. For each entry the dominant formation pathway, its principal molecular impact on NOX/NOS (activation, uncoupling or covalent modification) and a typical pathological or biomarker context are summarised.

While S-nitrosothiols such as GSNO or SSNO^−^ can inhibit NOX2 activation through the S-nitrosylation of regulatory subunits (primarily p47 phox, and potentially others such as p22 phox) their inhibitory effect on constitutively active NOX4 is limited due to its assembly-independent structure [169,170]. Furthermore, chronic oxidative stress impairs the function of thiol-dependent antioxidant systems, including thioredoxin (TRX. mainly TRX1), peroxiredoxin (PRDX. mainly PRX1/3), and glutaredoxin (GRX), largely due to NADPH and GSH depletion [171]. Consequently, this metabolic insufficiency not only undermines the redox-buffering capacity of S-nitrosothiols but may also favor the shift toward peroxynitrite (ONOO^−^) formation, thereby amplifying nitrative stress.

Abbreviations: HOCl, hypochlorous acid; HOBr, hypobromous acid; GSNO, S-nitrosoglutathione; IsoLG, isolevuglandin; AGE, advanced glycation end-product; ALE, advanced lipid-peroxidation end-product; MetS, metabolic syndrome; MASLD, metabolic-dysfunction- associated steatotic liver disease; BBB, blood–brain barrier. RAGE, receptor for advanced glycation end-products; AGE/RAGE pathway, advanced glycation end-product–RAGE signaling pathway.

#### 2.2.2 Reactive end-products that amplify and report NOX–NOS crosstalk

As described in Section 2.2.1 and Table 3, reactive intermediate metabolites and some enzymes associated with metabolic disorders and oxidative stress interact significantly with NOX and NOS. Reactive intermediate metabolites undergo several complex metabolic processes to form stable end-products, including advanced glycation end-products (AGEs), advanced lipid-peroxidation end-products (ALEs), and advanced oxidation protein products (AOPPs) [172]. Unlike short-lived intermediates, these macromolecular adducts persist within specific tissues and subcellular compartments, thereby amplifying chronic redox imbalance along the liver–brain axis.

AGEs are mainly generated through the non-enzymatic glycation of proteins, lipids, and nucleic acids with reducing sugars and their reactive carbonyl intermediates—such as methylglyoxal (MGO), glyoxal (GO), and 3-deoxyglucosone (3-DG)—under hyperglycemic conditions. These dicarbonyl compounds act as potent precursors that rapidly form advanced glycation end-products (AGEs), which are chemically stable, poorly degradable, and tend to accumulate in tissues exposed to metabolic stress [173]. Binding of AGEs to RAGE (the AGE/RAGE pathway) activates downstream signaling cascades, including NF-κB and MAPK pathways, which subsequently stimulate pro-inflammatory cytokine production and enhance oxidative stress through NOX activation. In fact, RAGE (receptor for AGE) pathway was found to upregulate NOX2 by promoting the phosphorylation of p47 phox in skeletal muscle cells [174], and the relationship between the RAGE pathway activation induced by STZ (streptozotocin) and the upregulation of p47 phox/NOX4 was explained in a preclinical diabetic rat model [175]. It was also demonstrated that MGO-derived AGEs (the MGO/RAGE pathway), formed through non-enzymatic glycation by the MGO, act as a major pathogenic factor in diabetic nephropathy by upregulating NOX4 expression and activating the NLRP3 inflammasome in diabetic animal models [149]. In the context of neurodegenerative disease, AGEs have also been shown to upregulate iNOS expression in Alzheimer's disease, thereby contributing to chronic nitrosative stress and neuroinflammation [176]. The findings imply that these intermediate metabolites and final metabolites are important links in establishing the NOX-NOS crosstalk between metabolism-redox-inflammation.

ALEs are major end-products of lipid peroxidation and include structurally diverse adducts formed through covalent modification of proteins by reactive carbonyl species (RCS) derived from unsaturated fatty acids and their metabolic intermediates [177]. Key precursors include 4-hydroxy-2-nonenal (4-HNE), malondialdehyde (MDA), and acrolein, which are generated during the peroxidation of ω-6 polyunsaturated fatty acids such as linoleic acid and arachidonic acid. These RCS react with nucleophilic residues on proteins to form a variety of ALEs [139]. In the context of the liver–brain axis, ALEs compromise protein integrity and signaling, promoting inflammation and metabolic dysfunction that underlie tissue damage across peripheral and central systems. 4-HNE is disrupts lipid metabolism by modifying apolipoprotein B and apolipoprotein E, and binds to neuronal membrane proteins, contributing to neurotoxicity and cognitive impairment [132]. Within mitochondria, 4-HNE can accumulate in the inner membrane and form covalent adducts with cardiolipin-bound proteins, impairing electron transport chain (ETC) function and ATP production [178]. MDA similarly forms adducts with mitochondrial cardiolipin-protein complexes, compromising ETC activity and bioenergetic efficiency. Its accumulation has been implicated in vascular and neuronal injury in models of metabolic stress [179]. Acrolein is often produced in the CNS via polyamine oxidation under oxidative stress conditions and is therefore considered a strong candidate contributing to the development of neurodegenerative diseases [141]. It localizes to mitochondria where it alkylates cysteine residues in ETC complexes, disrupts ATP synthesis, and enhances ROS generation. Acrolein also induces mitochondrial membrane depolarization and depletes mitochondrial glutathione reserves, amplifying oxidative damage. Additionally, it forms DNA-protein cross-links that contribute to genotoxicity and neuroinflammation [142]. Thus, the accumulation of ALE can be used as a biomarker reflecting chronic exposure to oxidative stress and metabolic abnormalities, and is closely related to the progression of various chronic diseases such as cardiovascular disease, neurodegenerative disease, and diabetes [180].

AOPPs occur when circulating proteins (particularly albumin) become “over-oxidized” by powerful oxidizing agents rather than by a single specific chemical reaction [181]. This is not a fixed product with a single chemical structure, but is considered to be the final oxidation product of proteins as a mixture of various oxidized modified proteins [182]. AOPP is mainly produced by the oxidation of proteins such as plasma albumin by oxidative stress, and, *in vivo*, the myeloperoxidase (MPO)–HOCl axis is the major pathway for it. MPO released from activated neutrophils and monocytes uses H_2_O_2_ and Cl^−^ to generate hypochlorous acid, which chlorinates tyrosine and oxidises side chains, producing dityrosine cross-links and carbonyl adducts on plasma proteins [[127], [183]]. Within the liver–brain axis, hepatic metabolic dysfunction and chronic NOX-driven oxidative stress can dysregulate innate immune responses by enhancing MPO and iNOS activity, thereby elevating systemic AOPP levels [117]. Circulating AOPPs can cross compromised Blood-Brain Barrier (BBB) regions or indirectly trigger central inflammation via RAGE and TLR4 activation in cerebral endothelial cells or microglia [184]. They are considered terminal oxidation products of proteins and reflect cumulative protein damage under chronic redox stress [185].

### 2.3 Mitochondria as a potential amplifier in NOX–NOS crosstalk

Beyond their canonical role in energy production, mitochondria are increasingly recognized as critical amplifiers and signal propagators in redox biology. In the context of NOX–NOS crosstalk, mitochondrial reactive oxygen species (mtROS) act as key modulators of oxidative stress dissemination throughout the cell and across tissues [186]. While the localization of NOX4 to mitochondria remains under debate, emerging evidence supports the notion that mitochondria may bidirectionally interact with NOX4 to modulate ROS production [187]. As discussed in Sections 2.2.1, 2.2.2, reactive intermediates and end-products may enhance NOX activity and contribute to the amplification of redox imbalance, BH_4_ depletion, and NOS uncoupling along the liver–brain axis. In this phased progression, mitochondria are likely to serve not only as redox-sensitive hubs but also as active participants in feedforward loops that exacerbate oxidative stress and facilitate inter-organ redox signaling along the liver–brain axis.

### 2.4 Importance of biomarkers in NOX and NOS activity

Biomarkers reflecting the activity of NOX and NOS provide valuable insights into systemic and localized oxidative stress, inflammation, and disease progression [188,189]. These biomarkers hold great potential as valuable tools for assessing disease progression, oxidative stress burden, and the efficacy of therapeutic interventions. However, current NOX- and NOS-related biomarkers face several limitations. For NOX, markers such as H_2_O_2_ and NADPH consumption rates are indirect and lack specificity or sensitivity for reliable application in biological systems [114]. Similarly, NOS-related markers, including nitrite/nitrate and citrulline, are influenced by multiple pathways, which may complicate interpretation and limit clinical utility [125], and methods that measure •NO in saliva, such as •NO strips, do not objectively reflect systemic levels [190]. To address these challenges, alternative oxidative stress-related markers may serve as useful complementary indicators. Markers such as protein carbonylation [[191], [192], [193]], native low-density lipoprotein (nLDL) [194], lectin-like oxidized low-density lipoprotein receptor-1 (LOX-1), oxidized LDL (oxLDL) [144], and 8-isoprostane may be indirectly referenced as supportive biomarkers for NOX-related oxidative stress, particularly under chronic metabolic dysfunction along the liver–brain axis [146,195].

Asymmetric dimethylarginine (ADMA) [196,197] and 3-nitrotyrosine [198] are commonly used as indicators of NOS inhibition and protein nitration, respectively, although they are not fully specific to NOS-derived pathways. Innovative approaches are advancing biomarker discovery. Omics technologies, including metabolomics and proteomics, provide a systemic understanding of oxidative stress and inflammation by identifying NOX- and NOS-related metabolites and modified proteins [133]. Imaging-based biomarkers generated by fluorescent probes and molecular imaging techniques, also provide non-invasive methods to visualize ROS and RNS activity in specific tissues [199]. Additionally, liquid biopsy technologies utilizing extracellular vesicles (EVs) containing NOX- and NOS-related proteins and metabolites provide a minimally invasive method to assess activity [200,201]. EVs have been demonstrated as potential biomarkers for neurodegenerative and other diseases, particularly the correlation between these diseases and oxidative stress [202,203]. These correlations highlight the utility of EVs in reflecting disease-related oxidative stress dynamics and in their application as reliable diagnostic tools. These emerging biomarkers and technologies have the potential to overcome existing limitations and improve the accuracy and applicability of NOX- and NOS-related biomarkers in clinical settings [204]. Plasma nitrate and nitrite levels have been associated with disease severity in various inflammatory conditions, including COVID-19 [205,206]. Although not specific to NOX or NOS, they may provide indirect insight into broader oxidative and nitrative stress, especially in settings of immune dysregulation and endothelial dysfunction.

## 3 NOX-NOS crosstalk in the liver-brain axis

This section outlines a three-phase model of NOX–NOS crosstalk along the liver–brain axis. We first describe the metabolic ‘trigger’ stage in the liver, where oxidative imbalance initiates NOX and NOS activation. Next, we explore the ‘fibro-sclerotic’ intermediate phase, characterized by persistent redox stress, eNOS uncoupling, and multi-organelle dysfunction. Finally, we detail the neurodegenerative phase, wherein redox escalation propagates through glia–neuron interactions, culminating in synaptic loss and neuronal death. These sequential stages provide a mechanistic framework for understanding redox-driven pathology across metabolic and neurodegenerative disorders.

### 3.1 Metabolic ‘trigger’ step initiating NOX-NOS crosstalk along the Liver–Brain axis

The liver serves as the central organ for cellular redox reactions. This process is operated by various antioxidant regulatory enzymes including SOD, mainly SOD1 and SOD2), catalase (CAT), peroxiredoxin (PRDX, predominantly PRDX1 and PRDX3), thioredoxin (TRX, mainly TRX1), and sulfiredoxin (SRX), as well as glutathione system regulatory enzymes such as glutathione peroxidase (GPx), glutathione reductase (GR), and glutathione S-transferase (GST) [207,208]. At the core of these processes are metabolic and antioxidant regulatory molecular mechanisms such as NRF2/ARE, LKB1/AMPK/PPARα, SIRT/PGC1α/UCP1, SREBP1c/PPARγ/C-EBPα/, and NQO1 [[209], [210], [211], [212]]. In liver diseases such as metabolic-associated steatohepatitis (MASH), metabolic dysfunction-associated steatotic liver disease (MASLD), and liver cirrhosis, the GSH-based redox homeostasis in the tissue is disrupted, and the liver is exposed to ROS- and RNS-mediated oxidative damage [[213], [214]]. This oxidative damage in the liver develops into systemic oxidative stress mediated by liver-derived O_2_•^-^/H_2_O_2_ and ONOO^−^ spill over systemically, with iNOS, uncoupled eNOS, and NOX1/2/4/5 acting as primary amplifiers [[118], [215], [216]]. Indeed, NOX and NOS serve as critical regulators of liver function, and play a central role in the development of liver diseases mediated by oxidative stress [[217], [218], [219], [220], [221], [222], [223]].

In MASLD, oxidative stress mediated by NOX in the liver induces systemic inflammation and metabolic dysregulation, exacerbating neuroinflammation in the brain [224,225]. Recent studies have found that MASLD is accompanied by increased systemic H_2_O_2_ levels, which activate microglia and astrocytes in the central nervous system (CNS), leading to neuroinflammation [[226], [227], [228], [229], [230]]. These findings strongly suggest the importance of targeting the NOX and NOS pathways in both diseases and homeostasis of liver and nervous tissue [231,232]. Activation of α1-adrenergic receptors upregulates NOX2, which in turn modulates hepatic metabolic pathways in concert with β-adrenergic receptor signaling [233]. Additionally, both iNOS, eNOS, and nNOS have been demonstrated to act as key regulators within the liver-brain axis in disease models [[234], [235], [236], [237]]. These insights reinforce the central role of the liver in systemic redox regulation and its potential to contribute to the progression of neurodegenerative disease via the liver-brain axis.

Fig. 2 synthesises the mechanistic links discussed above in a hepatocyte-centred schematic. It illustrates how (i) dyslipidaemia-induced NOX4 activation, combined with LKB1/AMPK suppression, impairs metabolic homeostasis; (ii) aldehydic “second hits” such as 4-HNE, acrolein, MGO/GO, and 3-DG exacerbate NOX–iNOS crosstalk via peroxynitrite formation; and (iii) progressive exhaustion of G6PD/NADK and LKB1/AMPK ultimately drives a fibro-sclerotic transition that feeds forward into endothelial dysfunction and neuroinflammation along the liver. This visual framework sets the stage for the following discussion on therapeutic leverage points targeting the NOX4–iNOS–NADPH axis. Together, the figure underscores the metabolic ‘trigger’ step that initiates NOX–NOS crosstalk and leads to a cascade of redox–inflammatory responses culminating in chronic liver–brain dysfunction.

### 3.2 Intermediate ‘fibro-sclerotic’ stage of the Liver–Brain axis

**Fig. 3:**
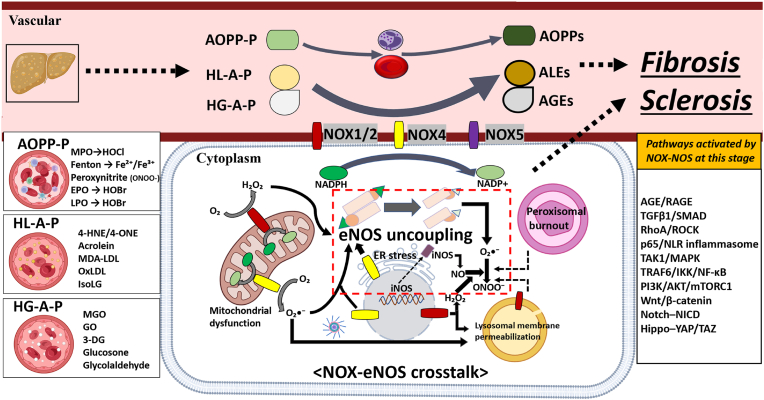
Intermediate “fibro-sclerotic” stage of the liver–brain axis: circulating R PAMPs prime NOX-driven eNOS uncoupling and multi-organelle stress. Reactive protein-adduct-making precursors (R PAMPs) in the bloodstream are subclassified into AOPP-P (AOPP precursors including HOCl, HOBr, Fe-Fenton reagents, and ONOO^−^), HL-A-P (high-lipid aldehyde precursors: 4-HNE/4-ONE, acrolein, MDA-LDL, oxLDL, IsoLG) and HG-C-P (high-glucose carbonyl precursors: MGO, GO, 3-DG, glycol-aldehyde). These ligands bind NOX1/2, NOX4, and NOX5 in the vascular endothelial cells and organ epithelial cells, and consume cytosolic NADPH, culminating in eNOS uncoupling. Consequent mitochondrial dysfunction, ER stress, lysosomal membrane permeabilisation and peroxisomal burnout amplify O_2_•^-^/H_2_O_2_ and ONOO^−^ release and activate profibrotic/prosclerotic pathways. The aggregate effect steers the axis toward tissue fibrosis and sclerosis, setting the stage for later neurodegeneration. These changes by the NOX-NOS crosstalk are important in regulating several related pathways to produce endothelial and organ fibrosis and sclerosis, and mediate the first steps leading to neurodegenerative disease development by compromising BBB integrity. Among the RCS, acrolein (56 Da), 4-HNE (156 Da), and others are able to pass through regardless of BBB integrity impairment and may mediate M1-like microglial activation in the CNS even before progression to chronic fibrosis and sclerosis through these pathways. Abbreviations: NOX, NADPH oxidase; NOS, nitric oxide synthase; eNOS, endothelial NOS; iNOS, inducible NOS; NADPH, nicotinamide adenine dinucleotide phosphate (reduced form); NADP^+^, oxidized NADPH; O_2_•^-^, superoxide anion; H_2_O_2_, hydrogen peroxide; ONOO^−^, peroxynitrite; AOPP, advanced oxidation protein product; ALE, advanced lipid peroxidation end-product; AGE, advanced glycation end-product; AOPP-P, AOPP precursor; HL-A-P, high-lipid aldehyde precursor; HG-A-P, high-glucose aldehyde precursor; HOCl, hypochlorous acid; HOBr, hypobromous acid; ONOO^−^, peroxynitrite; Fe^2+^/Fe^3+^, ferrous/ferric iron; 4-HNE, 4-hydroxy-2-nonenal; 4-ONE, 4-oxo-2-nonenal; MDA-LDL, malondialdehyde-modified low-density lipoprotein; oxLDL, oxidized low-density lipoprotein; IsoLG, isolevuglandin; MGO, methylglyoxal; GO, glyoxal; 3-DG, 3-deoxyglucosone; ER, endoplasmic reticulum; BBB, blood-brain barrier; RAGE, receptor for advanced glycation end-products; SMAD, mothers against decapentaplegic homolog; ROCK, Rho-associated coiled-coil-containing protein kinase; NLR, nucleotide-binding oligomerization domain-like receptor; TAK1, transforming growth factor beta-activated kinase 1; MAPK, mitogen-activated protein kinase; IKK, IκB kinase; NF-κB, nuclear factor-kappa B; PI3K, phosphoinositide 3-kinase; AKT, protein kinase B; mTORC1, mechanistic target of rapamycin complex 1; NICD, Notch intracellular domain; YAP/TAZ, Yes-associated protein/transcriptional co-activator with PDZ-binding motif.

Persistent hyperlipidaemia and hyperglycaemia caused by hepatic metabolic and redox imbalances set the stage for a second oxidative “hit” in which a heterogeneous pool of reactive protein-adduct-making precursors (R PAMPs) dramatically reshapes redox signaling. As previously mentioned in Section 2.1.2, metabolic syndrome is clearly associated with eNOS/nNOS uncoupling, and its close interaction with NOX is of critical importance. In particular, NOX4, an isoform that does not have a calcium EF domain, drives eNOS/nNOS uncoupling [238]. Fig. 3 depicts how prolonged NOX activation and accumulated aldehydes oxidise BH_4_, driving eNOS/nNOS uncoupling and amplifying ONOO^−^ production, thereby inaugurating the fibro-sclerotic stage and propagating neuroinflammation.” Plasma AOPP-P (HOCl/HOBr-modified albumin, Fe-catalysed carbonyls, ONOO^−^ adducts), HL-A-P (4-HNE/4-ONE, acrolein, MDA-LDL, IsoLG), and HG-A-P (MGO, GO, 3-DG, glucosone, glycolaldehyde) accumulate in proportion to metabolic burden and are readily taken up by hepatic sinusoidal endothelium, Kupffer cells, and brain microvascular endothelial cells [177−179]. These species act as DAMP-like ligands for NOX1/2 and NOX5, while simultaneously up-regulating NOX4 expression via ER-stress sensors (IRE1α/XBP1, ATF6) and the p65-NF-κB axis [239].

Inside the cytosol, hyper-activated NOX complexes siphon reducing equivalents from the NADPH/NADP^+^ pool, exhausting the G6PD-NADK salvage circuit and lowering the BH_4_:BH_2_ ratio. Oxidized BH_4_ fails to stabilize the NOS ferric-dioxy intermediate, converting eNOS from an •NO synthase to a superoxide-generating oxidase (eNOS uncoupling). The resulting O_2_•^-^/ONOO^−^ burst feeds forward to peroxisomal catalase inactivation and lysosomal membrane permeabilisation (LMP), liberating cathepsins that further cleave NOX regulatory subunits [[240], [241], [242]]. In parallel, mitochondrial complex I and III leak O_2_•^-^ under high-fat substrate pressure, amplifying the cytosolic H_2_O_2_ pool and activating the NLRP1/3/10 inflammasomes [243].

These redox-driven events converge on a shared set of fibro-sclerotic master pathways (Fig. 3), AGE/RAGE-p38 [244], TGF-β1/SMAD2-3 [245], RhoA/ROCK-MRTF-A [246], Wnt/β-catenin [247], Hippo-YAP/TAZ [248], and PI3K/AKT/mTORC1 [249]. Collectively they induce epithelial-to-mesenchymal transition (EMT), myofibroblast activation, and extracellular-matrix stiffening in liver and vascular beds. In the brain, low-molecular-weight aldehydes pass through the intact BBB; once BBB tight-junction integrity is compromised by oxidative stress, larger ALE/AGE aggregates traverse as well, provoking M1-like microglial activation and early synaptic pruning [250,140].

Importantly, circulating AOPP levels positively correlate with liver stiffness scores (transient elastography) and white-matter hyperintensity volume on MRI, suggesting that R PAMPs may serve as fluid biomarkers for the fibro-sclerotic transition [251,252].

### 3.3 Escalation of NOX–NOS crosstalk in the CNS and its convergence on neurodegeneration

**Fig. 4:**
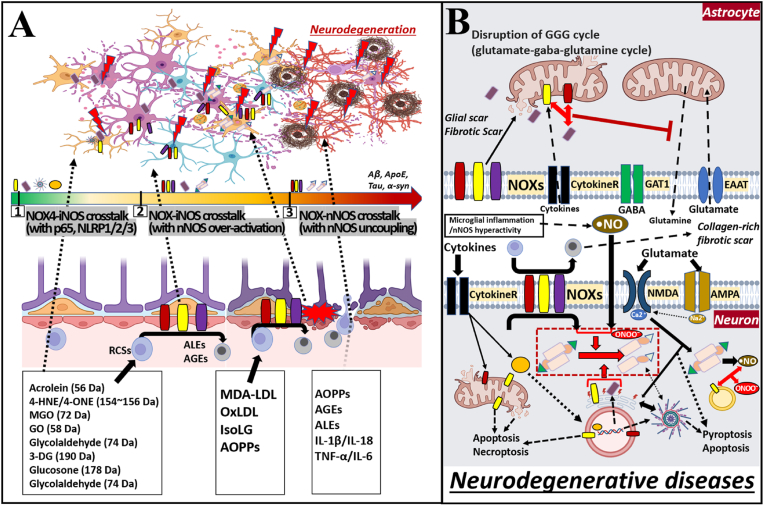
Phased escalation of NOX–NOS crosstalk in the CNS leading to neurodegeneration. (A) Timeline of redox escalation. (1) NOX4–iNOS crosstalk accompanied by p65-NF-κB and NLRP1/2/3 activation (green segment). (2) NOX–iNOS synergy + nNOS hyper-activation triggered by glutamate excitotoxicity (yellow). (3) NOX–nNOS uncoupling with ONOO^−^ amplification culminating in neuronal demise (red). (B) Astrocyte–neuron dyad at stage (3). NOX-rich astrocytic end-feet accumulate R PAMPs, secrete cytokines, and fail to recycle glutamine, disrupting the glutamate–GABA–glutamine (GGG) cycle. Excess synaptic glutamate opens NMDA/AMPA receptors, causing Ca^2+^ influx and nNOS activation/uncoupling in neurons. Intracellular O_2_•^-^ reacts with •NO to form ONOO^−^ (red dashed box), while mitochondrial, lysosomal, and ER stress drive apoptosis, necroptosis, and pyroptosis. Collagen-rich fibrotic and glial scars further isolate synapses and perpetuate redox imbalance. Abbreviations: NOX, NADPH oxidase; NOS, nitric oxide synthase; iNOS, inducible NOS; nNOS, neuronal NOS; eNOS, endothelial NOS; O_2_•^-^, superoxide anion; H_2_O_2_, hydrogen peroxide; ONOO^−^, peroxynitrite; •NO, nitric oxide; RCSs, reactive carbonyl species; R PAMPs, reactive protein-adduct-making precursors; ALEs, advanced lipid peroxidation end-products; AGEs, advanced glycation end-products; AOPPs, advanced oxidation protein products; MDA-LDL, malondialdehyde-modified low-density lipoprotein; oxLDL, oxidized low-density lipoprotein; IsoLG, isolevuglandin; MGO, methylglyoxal; GO, glyoxal; 3-DG, 3-deoxyglucosone; 4-HNE, 4-hydroxy-2-nonenal; 4-ONE, 4-oxo-2-nonenal; NF-κB, nuclear factor kappa-light-chain-enhancer of activated B cells; NLRP, NOD-like receptor family pyrin domain containing; NMDA, N-methyl-d-aspartate receptor; AMPA, α-amino-3-hydroxy-5-methyl-4-isoxazolepropionic acid receptor; GABA, γ-aminobutyric acid; GAT1, GABA transporter 1; EAAT, excitatory amino acid transporter; GGG cycle, glutamate–GABA–glutamine cycle; Ca^2+^, calcium ion; Na^+^, sodium ion; ER, endoplasmic reticulum; Apoptosis, programmed cell death; Necroptosis, programmed necrosis; Pyroptosis, inflammatory cell death.

Neuroglial cells orchestrate redox homeostasis and immune surveillance within the CNS [253]. Fig. 4 summarizes how successive waves of NOX–NOS dysregulation, seeded in the liver and vascular compartment, propagate through glia-neuron interactions to drive neurodegenerative pathology.

Phase 1 – NOX4-iNOS feed-forward loop in perivascular glia (green segment, Fig. 4). Reactive carbonyl species (RCS) generated in dysmetabolic liver—acrolein, 4-HNE/4-ONE, MGO, GO, glycolaldehyde, 3-DG—are small enough (≤200 Da) to traverse an intact BBB. These RCS, together with Kupffer-cell cytokines, activate NOX4 in astrocytic end-feet and perivascular microglia, inducing iNOS expression via p65-NF-κB and NLRP1/2/3 inflammasome priming [254]. Resultant H_2_O_2_ + •NO generate peroxynitrite (ONOO^−^), initiating the first NOX4-iNOS crosstalk node.

Phase 2 – NOX-iNOS synergy with nNOS hyper-activation in the tripartite synapse (yellow segment). Persistent ONOO^−^ oxidises BH_4_, depleting neuronal NADPH and lowering the BH_4_:BH_2_ ratio. Excitatory amino-acid transporters (EAATs) on astrocytes become carbonyl-modified, impairing glutamate uptake and disrupting the glutamate–GABA–glutamine (GGG) cycle [255,119]. Excess synaptic glutamate opens NMDA/AMPA receptors, allowing Ca^2+^ influx and CaM-dependent nNOS over-activation in neurons [256]. Concurrent M1-like activation of microglia sustains cytokine release (IL-1β, TNF-α, MIP-1α), further stimulating NOX1/2/4/5 across glia and neurons [257].

Phase 3 – NOX-nNOS uncoupling and multi-organelle catastrophe (red segment). When R PAMPs expand to include ALEs/AGEs [258,259] (MDA-LDL, oxLDL, IsoLG, AOPPs) that breach the now-compromised BBB, NOX-derived O_2_•^-^/H_2_O_2_ consume residual NADPH, fully uncoupling nNOS and creating self-propagating O_2_•^-^/ONOO^−^ loops inside neurons [115,116]. Mitochondrial complex I/III leakage [260,261], ER Ca^2+^ store collapse [262], lysosomal membrane permeabilisation [263], and peroxisomal burnout converge [264] on apoptotic, necroptotic and pyroptotic death pathways. Astrocytic NOX5 and cytokine receptors promote glial- and fibrotic-scar formation that isolates glutamatergic terminals and locks in metabolic hypereactivity [265].

Collectively, these phased events establish a chronic meta-inflammatory microenvironment in which redox-imbalanced astrocytes under-supply GSH (via GCL down-regulation), overproduce ROS/RNS, and fail to recycle neurotransmitters. Microglia transition to a phagocytic M1-like state but accumulate injurious AOPPs and IsoLG adducts that limit debris clearance [130]. Neurons, deprived of antioxidant support and exposed to sustained ONOO^−^, undergo synaptic loss, cytoskeletal collapse, and ultimately degenerative cell death. Thus, targeting stage-specific NOX/NOS nodes—NOX4-iNOS initiation, nNOS hyper-activation, and NOS uncoupling—offers a rational, time-stratified therapeutic strategy within the liver-brain axis framework [[266], [267], [268], [269], [270], [271], [272], [273]].

## 4 Translational opportunities—from mechanism to medicine: Therapeutic modulation and biomarker applications of NOX/NOS crosstalk

### 4.1 Crosstalk between NOX and NOS in liver-brain axis: application to current drug development

#### 4.1.1 Preclinical & clinical NOX/NOS inhibitors

Therapeutics targeting NOX and NOS have attracted attention as potential interventions to ameliorate these processes [[274], [275], [276]]. These therapeutic agents are summarized in Table 4, which outlines representative NOX and NOS modulators along with their proposed mechanisms and disease contexts. NOX inhibitors such as setanaxib (GKT137831) have shown efficacy in preclinical liver‐fibrosis and neuroinflammation models by reducing O_2_•^-^/H_2_O_2_ and restoring redox balance [156]. The NOX2 inhibitor GLX7013170 increased BCL-2 levels and augmented the expression of excitatory amino acid transporter 1 (EAAT1) in the retina of a diabetic retinopathy animal model, restoring glutamate metabolism and suppressing excitotoxicity and neuronal apoptosis [157]. Similarly, selective NOS inhibitors, particularly those targeting iNOS, are being explored for their ability to reduce RNS-mediated damage without Impairing the physiological functions of nNOS and eNOS [277,278]. Compounds like 1400W and L-NIL (L-iminoethyl-l-lysine) have shown promising effects in reducing iNOS activity and mitigating inflammation in both hepatic and neurological contexts [279,158]. N-phenylpropenoyl-1-1-amino acid (NPA) has also been identified as an promising drug candidate for Parkinson's disease as an iNOS inhibitor [159]. Furthermore, combination therapies targeting both NOX and NOS pathways have attracted much anticipation and are currently under investigation. For example, diphenyleneiodonium (DPI) has been reported to inhibit both NOX and iNOS [160], thereby suppressing β-amyloid deposition and alleviating Parkinson's disease as well as ischemic stroke [[280], [281], [282]]. Apocynin has also been shown to modulate both NOX and iNOS, demonstrating potential in improving ischemic stroke [161]. Drugs targeting the Fyn/SFK-iNOS-NOX2 signaling pathway exhibited dramatic activity to regulate the NOX-NOS axis [283]. This pathway is known to control the development of neurodegenerative and liver diseases by modulating microglial M1-like activation and the glutathione system [284,285]. Therefore, from the perspective of functional interactions along the liver-brain

**Table 4:** Current status of drug development targeting NOX or NOS.

Agent	Target	Key Models	Effect	Phase & Trial-ID	Ref
Setanaxib	NOX1 NOX4	MASH, NASH, stroke	↓ ROS, ↓ fibrosis	Ph II completed NCT03226067 (PBC) NCT05014672 (PBC)	[156]
GLX7013170	NOX2	Diabetic retinopathy	↑ BCL-2, ↑ EAAT1, ↓ apoptosis	Pre-clinical (Retinopathy)	[157]
1400W, L-NIL	iNOS	Hepatic/CNS injury	↓ RNS, ↓ inflammation	Pre-clinical	[158]
NPA	iNOS	Parkinson's model	Neuroprotection	Pre-clinical (PD)	[159]
DPI	NOX iNOS	AD & PD models	↓ Aβ, ↓ infarct size	Pre-clinical	[160]
Apocynin	NOX iNOS	Stroke	↓ NOX-p47phox, ↓ catalase	Ph I completed NCT03680638 (COPD) NCT01402297 (Skin Blood Flow)	[161]
APX-115	PAN NOX	Platelet aggregation & thrombosis models	↓ O_2_•^-^/H_2_O_2_ in platelets, ↓ SYK/LAT/PLCγ2, ↓ integrin αIIbβ3 activation, ↓ ATP release and P-selectin exposure	Ph II completed NCT04534439 (T2DM) Ph II Recruiting NCT05758896 (AKI)	[162]
VAS2870	PAN NOX	In vitro BBB model	↑ BBB integrity and function	Pre-clinical (BBB in vitro)	[163]
7-NI, l-NAME	nNOS	•NO-deficient hypertensive model	↑ SOD1 mRNA, ↑ SOD3, HO-1, AT2R, MDR1a mRNA	Pre-clinical (HTN)	[164]
GSK2795039	NOX2	Genetic hypertension model	↓ NOX2	Pre-clinical (HTN)	[165]
Sitagliptin	**DPP-4** NOX4	Streptozotocin induced diabetes model	↓ NOX4, ↓ MDA	**Approved (T2DM inhibitor)** Ph 1 (Unknown) NCT00696982 (HTN)	[166]
Gp91ds-tat	NOX2	CRS model	↓ NOX2, ↑ BDNF	Pre-clinical (CRS)	[167]
GLX7013159	NOX4 NOX2	Human islet–transplanted diabetic NMRI nu/nu mouse xenograft model	↓ NOX4, caspase-3	Pre-clinical (islet xenograft)	[168]

axis, the complex interplay of NOX and NOS in neurodegenerative diseases highlights the important role of the NOX-NOS axis and demonstrates its high potential as a therapeutic target [286].

Synthetic or repurposed pharmacological agents in clinical or late-stage pre-clinical development that directly inhibit NOX isoforms and/or iNOS. For each compound, the principal molecular target(s), key disease models, major redox/inflammatory outcomes, and supporting references are summarised. ↓ denotes inhibition or decrease; ↑ denotes activation or increase. *Abbreviations: MASH, metabolic dysfunction-associated steatohepatitis; NASH, non-alcoholic steatohepatitis; AD, Alzheimer's disease; PD, Parkinson's disease; BBB, blood–brain barrier; PAN NOX, pan-NADPH oxidase; nNOS, neuronal nitric oxide synthase; iNOS, inducible nitric oxide synthase; CNS, central nervous system; COPD, chronic obstructive pulmonary disease; ROS, reactive oxygen species; RNS, reactive nitrogen species; SYK, spleen tyrosine kinase; LAT, linker for activation of T cells; PLCγ2, phospholipase C gamma-2; AKI, acute kidney injury; ATP, adenosine triphosphate; SOD1, superoxide dismutase 1; HO-1, heme oxygenase 1; AT2R, angiotensin II type 2 receptor; MDR1a, multidrug resistance protein 1a; MDA, malondialdehyde; BDNF, brain-derived neurotrophic factor; CRS, chronic restraint stress; NMRI, Nuclear Magnetic Resonance Imaging*.

#### 4.1.2 State of the potential for natural Product–Based development

Natural products are a source of a wide variety of compounds with diverse structural properties and biological activities. Considering the facts mentioned in Section 2.4, natural compounds with clear chemical structures may be promising modulators for NOX and NOX-targeting therapeutic approaches. In fact, natural products containing a variety of bioactive compounds offer promising avenues for targeting the complex crosstalk between NOX and NOS (Table 5). These compounds may address the interconnected effects of oxidative damage induced by NOX and NOS by modulating both oxidative stress and inflammatory signaling pathways [287]. For example, cannabis-derived cannabidiol (CBD), an ingredient derived from *Cannabis Sativa* L, can effectively inhibit the NOX-NOS pathway [288]. This compound has also been shown to attenuate lipopolysaccharide (LPS)-induced NADPH oxidase activity in microglia by reducing intracellular NADPH synthesis [289]. Additionally, *in vivo* studies have demonstrated that CBD reduces hepatic expression of NOX2 isoforms (p67phox/gp91phox) and alleviates nitrosative stress in ethanol-induced models of alcoholic liver disease (ALD) [290]. These findings highlight the ability of CBD to modulate oxidative stress induced by NOX and NOS in both hepatic and neural tissues [291,292].

**Table 5:** Natural product-derived modulators of the NOX–NOS crosstalk.

Agent	Target	Key Model(s)	Effect	Ref
***Cannabidiol (CBD)***	NOX2, iNOS	LPS-activated microglia, ethanol-induced ALD mice, primary hepatocytes	↓ NOX2 activity & intracellular NADPH ↓ iNOS-derived RNS ↓ hepatic & neural nitro-oxidative stress	[[288], [289], [290], [291], [292]]
***Curcumin***	NOX, iNOS	Chronic stress models, LPS-stimulated microglia	↓ NOX-mediated O_2_•^-^/H_2_O_2_ ↓ iNOS ↓ Neuro-inflammation	[[293], [294], [295], [296]]
***Resveratrol***	NOX2, •NOX4, iNOS	Alcoholic liver disease, EAE/encephalomyelitis, subarachnoid hemorrhage, stroke/TBI, obese-mouse brain	↓ NOX/iNOS ↑ AMPK–SIRT1 axis ↑ SOD & GSH ↓ Lipid peroxidation, neuroprotection	[[297], [298], [299], [300], [301], [302]]
***Epigallocatechin gallate (EGCG)***	NOX2, iNOS	HAPI neurons & microglia, CCl_4_-liver fibrosis, Parkinson's, traumatic brain injury	↓ iNOS & TNF-α ↓ p47^phox translocation → •NOX2 inhibition ↓ Lipid peroxidation, anti-ferroptosis	[[303], [304], [305], [306]]
***Quercetin***	iNOS, HO-1, NQO-1	Endosulfan-induced neurotoxicity, M1-like activation of macrophages/microglia	↓ iNOS & M1-like activation ↑ Antioxidant enzymes, neuro-/anti-inflammatory protection	[[120], [307], [308]]

Other notable natural products include curcumin, resveratrol, epigallocatechin gallate (EGCG), and quercetin, each of which exhibit unique mechanisms of modulating NOX and NOS activity. Curcumin, a polyphenol extracted from turmeric, has been shown to downregulate NOX and iNOS in stress models and microglial inflammation models, suggesting its potential to control the NOX-NOS axis [[293], [294], [295], [296]]. Resveratrol, found in grapes and berries, ameliorates alcoholic liver disease through the AMPK/SIRT1/p38 MAPK pathway [297], and improves encephalomyelitis by inhibiting NOX2, NOX4, iNOS, and inflammatory cytokines [298]. It has also been proven to have a protective effect against subarachnoid hemorrhage by suppressing microglia cell activation [299]. Resveratrol also demonstrates neuroprotective activity against stroke and traumatic CNS injury by increasing SOD and GSH activity while inhibiting iNOS [300,301]. Furthermore, it significantly reduces lipid peroxidation in the brain tissue of obese mice [302]. EGCG, a catechin found in green tea, has been reported to improve stress-mediated inflammation by decreasing iNOS and TNF-α in HAPI rat hippocampal neurons and microglia [303]. In addition, it ameliorates liver injury and fibrosis via inhibiting lipid peroxidation and enhancing GPx activity [304], thereby preventing ferroptosis. These findings suggest a possibility that EGCG may play a protective role in oxidative stress-related brain diseases. Actually, EGCG was shown to prevent Parkinson's disease through inhibition of ferroptosis [305], and attenuated traumatic brain injury by suppression of NOX2 activation via inhibition of p47phox translocation [306].

Another promising natural antioxidant, quercetin, has also been shown to significantly mitigate neurotoxicity and neurodegeneration induced by endosulfan exposure in rats. Quercetin exerts its protective effects by regulating multiple antioxidant, inflammatory, and metabolic pathways, including iNOS, HO-1, and NQO-1 [120]. Additionally, quercetin inhibits M1-like activation in macrophages and microglia, contributing to its anti-inflammatory and neuroprotective properties [307,308].

Although natural compounds exhibit high potential in antioxidant, anti-inflammatory and metabolic regulation, their therapeutic applications are often limited by issues related to bioavailability and *in vivo* stability [309]. However, these limitations can be overcome through advanced delivery systems such as nanoparticle-based carriers, liposomal formulations, hydrogel-based systems, and multiomics technologies [[310], [311], [312], [313]]. If these strategies are effectively implemented, the development of NOX-NOS axis-targeted therapeutics based on single natural compounds is expected to be more feasible and clinically beneficial [[314], [315], [316]].

Representative natural compounds shown to attenuate NADPH-oxidase (NOX) and/or nitric-oxide-synthase (NOS) activity. For each agent, the predominant molecular target(s), key pre-clinical disease models, principal antioxidant/anti-inflammatory effects, and supporting references are summarised. ↓ indicates inhibition or decrease; ↑ indicates activation or increase.

ARE, antioxidant-response element; BH_4_, tetrahydrobiopterin; DMF, dimethyl fumarate; GABARAPL1, γ-aminobutyric-acid-receptor-associated protein-like 1; GCL, glutamate-cysteine ligase; GSH, reduced glutathione; GR, glutathione reductase; HO-1, heme oxygenase-1; MASLD, metabolic dysfunction-associated steatotic liver disease; NOX, NADPH oxidase; NOS, nitric-oxide synthase; NRF1/2, nuclear-factor erythroid-derived 2-related factors 1/2; NQO1, NAD(P)H quinone oxidoreductase-1; NLRP, nucleotide-binding oligomerization domain-like receptor pyrin domain; ONOO^−^, peroxynitrite; p62/SQSTM1, sequestosome-1; ROS, reactive oxygen species; RNS, reactive nitrogen species; SOD2, superoxide dismutase 2.

### 4.2 Crosstalk between NOX and NOS in liver-brain axis*:* clinical Application along disease chronology

#### 4.2.1 Therapeutic positioning of NOX–NOS crosstalk: Rationale for stage-specific strategies

In sections 2.4, we discussed potential indicators of NOX and NOS activity. NOX and NOS are closely related to the pathogenesis of neurodegenerative diseases, and are therefore very promising targets for therapeutic strategies for these diseases. However, although NOX-targeting therapies can play a pivotal role, they often face limitations in the translational process, such as low target specificity, off-target effects, and variability in tissue expression profiles [317]. Similarly, NOS enzymes, especially eNOS, are indicators that reflect the influence of various physiological states such as vascular tone, aging, and metabolic flexibility, so it can be difficult to find a reliable ‘middle point’ that can be reflected as an indicator of disease state [318]. In fact, nitric oxide levels, which are direct indicators of NOS, are mostly used in aging and the need for physical activity [319].

In this review, we suggest that the perspective of the liver-brain axis can be a good hint as a reference point to approach these limitations. That is, from the perspective of the liver-brain axis, these limitations highlight the need for a therapeutic perspective specific to the disease stage [320]. As previously discussed, during the sequential progression of NOX–NOS crosstalk along the liver–brain axis, eNOS uncoupling acts as the primary mediator in the intermediate stage, whereas nNOS uncoupling (primarily within CNS cells) acts as the primary mediator in the terminal phase. These findings imply that careful consideration is needed to determine whether targeting only NOX in the treatment of neurodegenerative diseases would be beneficial, or whether targeted therapies that take both NOX and NOS into account would be effective.

#### 4.2.2 Suggestions for practical clinical applications of NOX-NOS crosstalk: early-phase NOX-focused therapy

Because redox and metabolic dysregulation in the early stages of disease progression remains potentially reversible, this window offers a critical opportunity for intervention. Intervention at the early phase of NOX–NOS crosstalk along the liver–brain axis shares key principles with therapeutic strategies for early metabolic disorders.

In the early stage of metabolic diseases characterized by transient oxidative bursts or mitochondrial dysfunction, NOX-derived O_2_•^-^/H_2_O_2_ may be the main cause, and therefore primary inhibition of NOX is necessary to prevent these diseases [321]. In addition, various factors that indicate oxidative status, such as glutathione levels and the ratio of NADPH and NADP, can be applied, and also, depending on the subject's environmental characteristics of metabolic diseases, serological indicators such as ALT, AST, γ-GTP and glycated hemoglobin (HbA1c), can be considered simultaneously [322]. Indeed, the combination of these diverse factors could be a very promising indicator for therapeutic approaches to NOX-mediated diseases. Although HbA1c does not directly quantify individual reactive dicarbonyls, its interpretation alongside circulating MDA, methylglyoxal, and 8-isoprostane yields an integrated “glyco-oxidative burden” profile that more accurately captures cumulative redox stress in type 2 diabetes [323,324]. And in patients with type 2 diabetes who have relatively low GSH and NADPH levels and high MGO and GO levels, the use of NOX inhibitors in combination with basic drug guidelines such as metformin may sufficiently contribute to the prevention of chronic diabetes and secondary complications [[325], [326], [327]].

#### 4.2.3 Suggestions for practical clinical applications of NOX-NOS crosstalk: chronic-phase dual NOX–NOS strategy

In patients with chronic metabolic diseases, such as type 2 diabetes patients with hyperlipidemia or dyslipidemia, or patients with hepatic fibrosis and atherosclerosis, it can be recommended that dual targeting of NOX and NOS be actively considered to restore redox homeostasis in addition to the treatment of metabolic diseases [[328], [329], [330]]. In cases with blood lipid abnormalities or obesity, MGO and GO are good indicators, and oxLDL, acrolein, and 4-HNE can also be considered as indicators [331,332]. Furthermore, well-known metabolic abnormality indicators such as blood triglycerides, cholesterol ratio, and apolipoprotein B can be helpful in classifying patients according to the chronicity of the disease [333,334]. In addition to metabolic indicators, inflammation levels can also be important clues. For example, in patients with metabolic syndrome with •NO specific underlying diseases, MPO should be actively considered as a predictive indicator if CRP levels are observed to be somewhat high [335,336]. In fact, MPO is significantly secreted by CRP in immune cells and is an indicator that strongly contributes to the development of atherosclerosis. As described above, MPO (when abnormally produced/functioning) contributes to eNOS/nNOS uncoupling through extensive interactions with NOX in the liver-brain axis and is an important indicator that induces M1-like activation of microglia in the CNS. In chronic diseases, M1-like activation associated with increased MPO occurs in macrophages and microglia [337,338]. Therefore, in patients with chronic immune diseases such as atopic dermatitis, measuring MPO together with CRP may serve as a key judgment indicator for NOX-NOS-related therapeutic approaches in the presence of liver metabolic diseases.

Thus, we propose that approaches targeting NOX and NOS—as central mediators of chronically induced redox reactions within the liver–brain axis—could offer significant potential and insights for therapeutic strategies that integratively address redox, metabolic, and immune dimensions.

### 4.3 Crosstalk between NOX and NOS in liver-brain axis: pathway-targeted modulation of the NOX–NOSs

Building on biomarker-guided stratification, another promising avenue lies in targeting regulatory pathways associated with NOX–NOS crosstalk. Table 6 organises these 'master pathways' step by step and axis by axis to provide a map for future targeted therapeutic strategies. Among the many signaling-related molecules, NRF2 plays a central role in both liver and nervous tissue through activation of the expression of antioxidant enzymes such as HO1, NQO1, and GCLM. [339,340]. During the development of neurodegenerative diseases, disruption of the NRF2/HO-1 and NRF2/ARE signaling pathways aggravates oxidative stress and neuroinflammation [341,342]. Equally, in liver dysfunctions such as MASH, excessive O_2_•^-^/H_2_O_2_ and ONOO^−^, production by NOX and NOS, along with lipid peroxidation-induced ferroptosis, may overwhelm antioxidant defense systems including glutathione, resulting in insufficient NRF2 activation in neural tissues [343,344]. Dimethyl fumarate (DMF), a clinically approved NRF2 activator, has been shown to be effective in mitigating oxidative damage caused by NOX and NOS [345]. Additionally, NRF2 activators such as bardoxolone methyl are also attracting attention as drugs that can suppress hepatic and neuronal diseases associated with ROS and RNS. [[346], [347], [348]].

**Table 6:** Master pathways that can be therapeutically leveraged to modulate the NOX–NOS crosstalk along the liver–brain axis.

Lever Point/Axis	Core Mechanism (liver–brain axis relevance)	Representative Modulators	Key Evidence Snapshot	Refs
**NRF2-driven antioxidant induction**	NRF2 → ARE transcription ↑ → HO-1, NQO1, GCLM, SOD2 ↑ → boosts GSH pool, quenches O_2_•^-^/H_2_O_2_ and ONOO^−^, → lowers NOX4 output & iNOS induction in hepatocytes + glia	**Clinical/late-stage:** dimethyl fumarate (DMF), bardoxolone methyl **Pre-clinical/dietary:** sulforaphane, oltipraz	DMF dampened NOX2 and iNOS in MPTP mouse brain (↓ONOO^−^, ↓microglial activation) Bardoxolone reduced hepatic O_2_•^-^/H_2_O_2_ and improved MASLD scores in phase 2 trial Sulforaphane lowered NOX4/iNOS mRNA in HFD-rat cortex	[253−256]
**NRF1/p62-mediated autophagy enhancement**	NRF1 activation → p62 → GABARAPL1-dependent selective autophagy → clears oxidized proteins & NLRP-inflammasomes → interrupts NOX–iNOS feedback	tert-butylhydroquinone (TBHQ), tangeretin, oltipraz, exercise-mimetic AMPK activators	p62 transgenic mice resist iNOS up-regulation during LPS challenge (microglia + hepatocytes) TBHQ restored p62 and reduced NOX4 protein carbonylation in MASLD model	[257−260]
**Glutathione-axis restoration (GSH/GCL/GR)**	Enhancing GCL (rate-limiting) or GR activity preserves NADPH & BH_4_ → prevents eNOS/nNOS uncoupling, stabilises mitochondrial redox	N-acetylcysteine (NAC), N-acetylcysteine amide, l-cysteine ethyl ester, α-lipoic acid; GCL inducers (such as fumarates), GR activators	NAC (1 g/day) lowered plasma AOPP & 3-NT in NASH patients NACA rescued BH_4_ levels, normalised cerebral blood flow in HFD mice	[310,311]

The NRF1/p62 pathway is increasingly recognized for its role in autophagy and cellular homeostasis. Since the NRF1/p62 pathway works as a mitigator of neurotoxicity, inflammatory responses, and Aβ deposition through GABARAPL1 activation, this pathway is attracting attention as an effective therapeutic target for neurodegenerative diseases [349,350]. NRF1 has been known to enhance GPx activity [351] while exhibiting inhibitory effects on ROS and iNOS [352,353]. Meanwhile, p62 is closely related to liver function and acts as a key regulator of NLRP3 inflammasome, so this molecule is considered to play an important role in liver-brain axis function [354]. N-acetylcysteine (NAC) and other GSH precursors have potent antioxidant activity and can restore glutathione homeostasis via the glutamate-cysteine metabolic pathway, thus serving as promising approaches to efficiently modulate liver-brain axis function [355]. Targeting the activation of key enzymes involved in GSH metabolism, such as glutamate-cysteine ligase (GCL) and glutathione reductase (GR), is consistent with this strategy to enhance GSH activity [356]. Based on the mechanisms discussed in Sections 2, 3, targeting reactive intermediates or end-products associated with mitochondrial damage represents a mechanistically grounded therapeutic strategy. For example, mitochondria-targeted IsoLG scavengers, such as mito-2-HOBA, may represent a promising strategy to ameliorate NOX- and NOS-related oxidative stress and metabolic dysregulation [143].

This table maps key “Lever Points/Axis” within the liver–brain axis, the core redox-metabolic mechanisms they engage in NOX–NOS crosstalk, representative modulators (clinical, preclinical, or dietary), and concise evidence of their efficacy. It is organized stepwise—first by upstream transcriptional control (NRF2-driven antioxidant induction), then by proteostatic maintenance (NRF1/p62-mediated autophagy), and finally by glutathione axis restoration (GSH/GCL/GR)—to illustrate master pathways for future therapeutic strategies. Each pathway interrupts the NOX–NOS feedforward loop at a distinct node: NRF2 activation boosts ARE-dependent enzymes (HO-1, NQO1, GCLM, SOD2) to quench O_2_•^-^/H_2_O_2_ and ONOO^−^, and limit NOX4/iNOS induction; NRF1/p62 clears oxidized proteins and inflammasomes via selective autophagy; and GSH axis enhancement preserves NADPH/BH_4_ to prevent NOS uncoupling and stabilize mitochondrial redox. Representative agents ranging from dimethyl fumarate and bardoxolone methyl to sulforaphane, TBHQ, and N-acetylcysteine have demonstrated efficacy in models of neurodegeneration, MASLD, and NASH [[339], [340], [341], [342], [343], [344], [345], [346], [347], [348], [349], [350], [351], [352], [353], [354], [355], [356]].

### 4.4 Translational challenges, limitations, and future directions

Despite the therapeutic promise of NOX- and NOS-directed agents, several critical translational hurdles remain. The structural diversity and isoform-specific regulatory mechanisms of both NOX and NOS complicate drug development, making it difficult to design compounds that selectively engage individual subunits.

Many currently studied NOX/NOS inhibitors also face important agent-specific limitations. DPI indiscriminately blocks all flavin-containing enzymes, including mitochondrial complex I and eNOS, resulting in cytotoxicity at micromolar concentrations. Apocynin shows limited isoform selectivity and poor oral bioavailability. Several iNOS inhibitors, such as 1400W or L-NIL, suffer from rapid metabolic clearance, while newer compounds like GLX7013170 lack sufficient pharmacokinetic data in humans. Sitagliptin, though widely prescribed as a DPP-4 inhibitor for type 2 diabetes, requires further mechanistic evaluation before it can be repositioned as a dual NOX/NOS modulator.

From a broader perspective, redox imbalance and metabolic dysfunction underlie a wide spectrum of chronic diseases, thereby supporting the potential of integrated therapeutic strategies. In this context, natural product-derived compounds, such as curcumin or resveratrol, exhibit considerable promise in modulating redox-inflammatory pathways. However, their generally low target specificity remains a key barrier to clinical translation. Nevertheless, given the broad and potent antioxidant activities of natural compounds, combinatorial strategies that employ NOS-targeting drugs as a foundation and integrate natural compounds to further inhibit NOX activity and NOX-derived ROS may offer promising therapeutic potential, particularly in the context of the liver–brain axis.

**Fig. 5:**
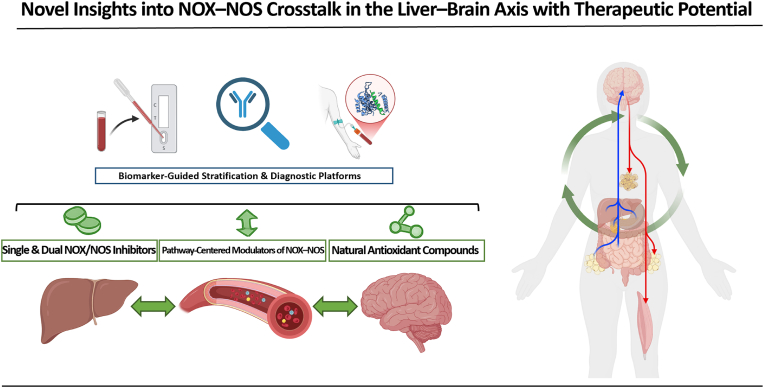
The Potential of NOX- and NOS-Targeted Therapeutic Strategy from the Perspective of the Liver-Brain Axis. By leveraging biomarker-based stratification and highly specific, efficient diagnostic platforms, three therapeutic strategies targeting NOX–NOS crosstalk can be concretely implemented. Restoring redox, metabolic, and immune homeostasis embodies healthy living within the liver–brain axis and broader multi-organ network paradigms.

Similarly, the successful development of drugs targeting redox and metabolic pathways must be supported by the parallel advancement of sensitive biomarker platforms and diagnostic technologies. Ultimately, the development of isoform-selective, bioavailable, and CNS-penetrant modulators remains essential for clinical translation along the liver–brain axis. Furthermore, future research directions should incorporate comprehensive *in vivo* validations alongside multi-omics strategies, including single-cell sequencing, spatial transcriptomics, and metabolomics. These advanced approaches will facilitate a deeper understanding of redox-metabolic heterogeneity across organ systems and disease stages, thereby enhancing the translational potential of NOX/NOS-targeted therapies (Fig. 5).

## 5 Conclusions

According to physical principles, matters tend to remain in its most stable state [357]. Therefore, redox homeostasis can be considered the closest approach to nature's fundamental principle for maintaining health in the body, and it is also a response that occurs in complex interrelationships with numerous diseases [358]. From a cellular biochemical perspective, the liver-brain axis can be defined as the result of a series of progressively strengthened biochemical interactions between the two organs that begin in the gut [359]. Within the cells of each tissue, these interactions would have been optimized for survival and would have been tightly integrated into metabolic regulation and neural control [360]. As a result, this system was defined by the modern concept of the liver-brain axis [361]. This approach may provide novel insights into the understanding of various diseases. Much like AMPK acts as a central regulator of various metabolic processes [362], NOX and NOS play pivotal roles in oxidative damage and subsequent inflammatory responses. In this review, we build on this perspective and discuss the interplay between O_2_•^-^/H_2_O_2_ production via NOX and ONOO^−^ production via NOS in the liver-brain axis, with a particular focus on their implications in neurodegenerative diseases.

In summary, along the liver–brain axis, NOX–NOS crosstalk propagates oxidative damage systemically via three sequential phases. First, the metabolic ‘trigger’ phase—mediated by NOX–iNOS crosstalk—features excessive hepatic generation of superoxide and H_2_O_2_, depleting NADPH and GSH pools and producing a variety of reactive metabolic intermediates. Second, during the ‘fibro-sclerotic’ phase, NOX–eNOS crosstalk from circulating intermediates activate endothelial NOX, further exhausting endothelial NADPH and driving chronic fibrosis and vascular sclerosis through eNOS/nNOS uncoupling. Third, in the ‘central overload/degeneration phase’ governed by NOX–iNOS–nNOS crosstalk, lipid peroxidation products (4-HNE, methylglyoxal, 3-deoxyglucosone) induce ferroptosis and pyroptosis in the CNS, culminating in neurodegeneration.

Because this organ-spanning cascade reflects an integrated redox collapse, the NADPH:NADP^+^ ratio—the definitive metric of cellular reducing power—emerges as a unifying diagnostic marker. Recent advances in real-time NADPH biosensing (such as NAPstar probes) and next-generation drug-delivery/biosensing platforms may not only quantify NOX–NOS-mediated oxidative injury in metabolic syndrome patients [363], but also substantially mitigate the challenges of therapeutically targeting the NOX–NOS axis even in the face of progressive liver dysfunction. Based on these concepts, we highlighted the importance of the ‘NOX–NOS axis’ as a core framework that provides new insights and approaches to the pathogenesis and therapeutic strategies of neurodegenerative diseases from the perspective of the liver–brain axis. Therapeutic strategies to block NOX and NOS activity using specific inhibitors have shown promising results in reducing oxidative damage, mitigating neuroinflammation, and preserving endothelial integrity.

Therefore, when all these results are combined, therapeutic approaches targeting the NOX-NOS axis, a key regulator of the complex crosstalk control among the three elements of metabolism, redox, and inflammation, may open new possibilities for the prevention and treatment of neurodegenerative diseases caused by crosstalk of the liver-brain axis.

## CRediT authorship contribution statement

**Sang-Seop Lee:** Writing – original draft, Visualization, Methodology, Investigation, Conceptualization. **Yung-Choon Yoo:** Writing – review & editing, Visualization, Supervision, Project administration.

## Declaration of competing interest

The authors declare that they have no known competing financial interests or personal relationships that could have appeared to influence the work reported in this paper.

## Figures and Tables

**Table:** 

4-HNE	4-Hydroxy-2-Nonenal
**ACC**	**Acetyl-CoA Carboxylase**
**ACSL4**	**Acyl-CoA Synthetase Long-Chain Family Member 4**
**AD**	**Alzheimer's Disease**
**AGEs**	**Advanced Glycation End Products**
**AKT**	**Protein Kinase B**
**Akt (*p*-Akt)**	**Protein Kinase B (Phospho-Protein Kinase B)**
**ALE**	**Advanced Lipoxidation End Products**
**ALS**	**Amyotrophic Lateral Sclerosis**
**AMPK**	**AMP-Activated Protein Kinase**
**AOPP**	**Advanced Oxidation Protein Products**
**ARE**	**Antioxidant Response Element**
**ATF4**	**Activating Transcription Factor 4**
**ATG7**	**Autophagy-Related Protein 7**
**BBB**	**Blood–Brain Barrier**
**BH4**	**Tetrahydrobiopterin**
**BIM**	**BCL-2-Interacting Mediator of Cell Death**
**CHOP**	**C/EBP Homologous Protein**
**cNOS**	**Constitutive Nitric Oxide Synthase**
**CNS**	**Central Nervous System**
**COX-2**	**Cyclooxygenase-2**
**CPT1**	**Carnitine Palmitoyltransferase 1**
**DPI**	**Diphenyleneiodonium**
**EGCG**	**Epigallocatechin Gallate**
**eNOS**	**Endothelial Nitric Oxide Synthase**
**Ero1α**	**Endoplasmic Reticulum Oxidoreductin 1α**
**FASL**	**Fas Ligand**
**G6PD**	**Glucose-6-Phosphate Dehydrogenase**
**GABARAPL1**	**GABA Type A Receptor-Associated Protein Like 1**
**GPx**	**Glutathione Peroxidase**
**GPX4**	**Glutathione Peroxidase 4**
**GR**	**Glutathione Reductase**
**GSH**	**Glutathione**
**GSK3β**	**Glycogen Synthase Kinase 3 Beta**
**GSSG**	**Oxidized Glutathione**
**H&E**	**Hematoxylin & Eosin staining**
**HO**_2_	**Hydrogen Peroxide**
**HFD**	**High-Fat Diet**
**HMOX1**	**Heme Oxygenase 1**
**IHC**	**Immunohistochemistry**
**IL-1β**	**Interleukin 1 Beta**
**iNOS**	**Inducible Nitric Oxide Synthase**
**IP**	**Immunoprecipitation**
**IR**	**Insulin Receptor**
**IRS-1**	**Insulin Receptor Substrate 1**
**JNK**	**c-Jun N-terminal Kinase**
**KEAP1**	**Kelch-Like ECH-Associated Protein 1**
**l-NAME**	**NG-Nitroarginine Methyl Ester**
**LOX-1**	**Lectin-Like Oxidized Low-Density Lipoprotein Receptor 1**
**LPS**	**Lipopolysaccharide**
**MASH**	**Metabolic Dysfunction-Associated Steatohepatitis**
**MASLD**	**Metabolic Dysfunction-Associated Steatotic Liver Disease**
**MDA**	**Malondialdehyde**
**MGO**	**Methylglyoxal**
**NADK**	**NAD Kinase**
**NASH**	**Non-Alcoholic Steatohepatitis**
**NFAT**	**Nuclear Factor of Activated T Cells**
**NF-κB**	**Nuclear Factor Kappa-Light-Chain-Enhancer of Activated B Cells**
**NLRP3**	**NLR Family Pyrin Domain Containing 3**
**nNOS**	**Neuronal Nitric Oxide Synthase**
**NOS**	**Nitric Oxide Synthase**
**NOX**	**NADPH Oxidase**
**NQO1**	**NAD(P)H Quinone Dehydrogenase 1**
**NRF1**	**Nuclear Factor Erythroid 2-Related Factor 1**
**NRF2**	**Nuclear Factor Erythroid 2-Related Factor 2**
**ONOO^−^**	**Peroxynitrite**
**p62**	**Sequestosome 1**
**p65 (RELA)**	**NF-κB Subunit**
***p*-ACC**	**Phospho-Acetyl-CoA Carboxylase**
**PD**	**Parkinson's Disease**
**PERK**	**PKR-Like Endoplasmic Reticulum Kinase**
**PGC-1α**	**Peroxisome Proliferator-Activated Receptor Gamma Coactivator-1α**
**PI3K**	**Phosphoinositide 3-Kinase**
**PKC**	**Protein Kinase C**
**PPARγ**	**Peroxisome Proliferator-Activated Receptor Gamma**
**PRX**	**Peroxiredoxin**
**PTP1B**	**Protein Tyrosine Phosphatase 1B**
**RAGE**	**Receptor for Advanced Glycation Endproducts**
**RNS**	**Reactive Nitrogen Species**
**ROS**	**Reactive Oxygen Species**
**SIRT1**	**Sirtuin 1**
**SLC7A11**	**Solute Carrier Family 7 Member 11**
**SOD**	**Superoxide Dismutase**
**SOD1**	**Superoxide Dismutase 1**
**SOD2**	**Superoxide Dismutase 2**
**STAT1**	**Signal Transducer and Activator of Transcription 1**
**TGF-β**	**Transforming Growth Factor Beta**
**TNF-α**	**Tumor Necrosis Factor Alpha**
**TRAIL**	**TNF-Related Apoptosis-Inducing Ligand**
**TRX**	**Thioredoxin**
**UCP1**	**Uncoupling Protein 1**

## Data Availability

No data was used for the research described in the article.
